# Intragenic regulation of SOCS3 isoforms

**DOI:** 10.1186/s12964-019-0379-6

**Published:** 2019-06-25

**Authors:** Oliver Klepsch, Lise Sarah Namer, Nadine Köhler, Raymond Kaempfer, Anna Dittrich, Fred Schaper

**Affiliations:** 1 Department of Systems Biology, Institute of Biology, Otto-von-Guericke University Magdeburg, Universitätsplatz 2, 39106 Magdeburg, Germany; 2 Department of Biochemistry and Molecular Biology, Institute of Medical Research Israel-Canada, The Hebrew University-Hadassah Medical School, 9112102 Jerusalem, Israel

**Keywords:** Interleukin-6, Signal transduction, SOCS, JAK, STAT, Stress response, PKR

## Abstract

**Background:**

Inflammatory reactions are commonly affected by stress responses. Interleukin-6 signalling is part of the inflammatory response and is stringently regulated by the feedback inhibitor SOCS3 expressed in a short and long isoform. Here, we studied the inhibitory potential of the two SOCS3 isoforms. Furthermore, we analysed the regulation of SOCS3 isoform expression and the role of PKR stress kinase signalling in SOCS3 protein expression.

**Methods:**

We performed Western blotting, reporter assays, genetic analyses and manipulations for studying SOCS3 isoform expression and activation of signalling components involved in interleukin-6-induced and PKR-dependent signalling.

**Results:**

Interleukin-6-induced endogenous expression of both SOCS3 isoforms was found in distinct cell types. Forced expression of either the long or short SOCS3 isoform demonstrated equal inhibitory activity of each isoform and confirmed longer half-life of the short isoform. Study of intragenic regulation of SOCS3 isoform expression revealed that (i) the 5′-UTR of *SOCS3* mRNA restrains specifically expression of the long SOCS3 isoform, (ii) expression of the long isoform restrains expression of the short isoform, and (iii) signalling through the stress kinase PKR does not impact on SOCS3 isoform ratio.

**Conclusions:**

Both SOCS3 isoforms show a similar potential for inhibiting interleukin-6 signalling but differ in their half-lives. Relative expression of the isoforms depends on intragenic elements yet is independent of PKR signalling.

**Graphic abstract:**

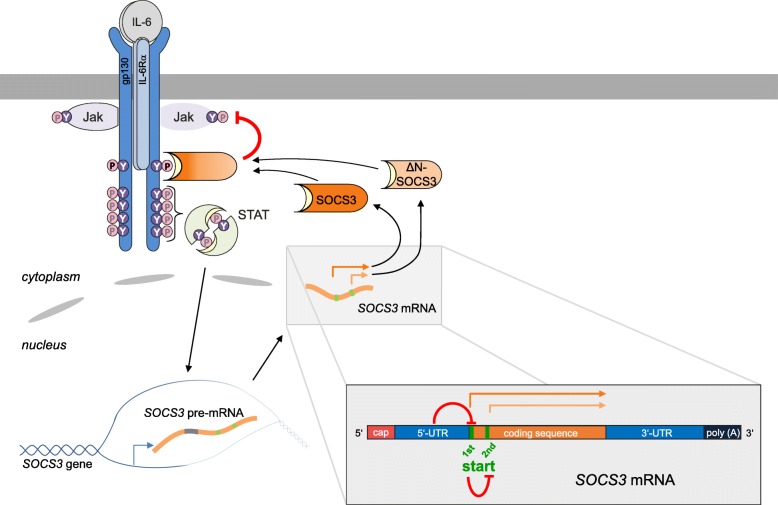

**Electronic supplementary material:**

The online version of this article (10.1186/s12964-019-0379-6) contains supplementary material, which is available to authorized users.

## Plain English summary

Inflammation is a defence mechanism of the organism to cope with infections and other traumata. Interestingly, inflammatory reactions are commonly affected by cellular stress responses. The tissue hormone interleukin-6 is an important regulator of the inflammatory reaction. As overshooting inflammation induces pathologies such as autoimmune diseases, chronic inflammation, and even cancer, the action of interleukin-6 is rigorously controlled. Our study deals with an important negative feedback inhibitor of interleukin-6 action, called SOCS3. SOCS3 exists in two isoforms of different length that are encoded by one gene. We asked how the ratio of these two isoforms is regulated, whether both isoforms have different regulatory potential, and whether their presence is regulated by stress stimuli. Our results show that both isoforms exert similar inhibitory power but one isoform is more stable than the other. Surprisingly, expression levels of the specific isoforms do not correlate with their specific stability but are influenced by regulatory elements within the gene coding for SOCS3. Stress stimuli do not affect the expression of both isoforms. Thus, the inhibitory potential of SOCS3 depends solely on the presence of either isoform. Our study may help to understand how inflammation is controlled via SOCS3.

## Background

Regulation of the inflammatory response by stress is a commonly observed phenomenon. At the cellular level, signalling induced by either stress hormones or intracellular stress sensors cross-talks with inflammatory cytokine signalling. For example, glucocorticoids that regulate glucose metabolism to increase blood glucose in stress situations also increase the hepatic expression of acute-phase proteins [1, 2]. Expression of these proteins is an important part of the acute-phase response and thus part of the systemic innate inflammatory response. The major inducer of acute-phase protein expression is the inflammatory cytokine interleukin-6 (IL-6) [3]. IL-6 activates the IL-6-receptor complex composed of the transmembrane and signal transducing subunit gp130 and either the transmembrane or the soluble IL-6 receptor α (IL-6Rα). Upon binding of IL-6 to the receptor complex, gp130-associated Janus kinases (JAK) are activated and subsequently phosphorylate tyrosine motifs within the cytoplasmic part of gp130 [4]. These phosphotyrosine motifs are recruitment sites for transcription factors of the signal transducer and activator of transcription (STAT) family. STAT factors become phosphorylated, dimerize and translocate into the nucleus where they activate transcription of IL-6-inducible genes, such as acute-phase genes [5, 6]. Necessarily, activation of the inflammatory response must be stringently controlled, negative regulation at the level of signal transduction being of paramount importance.

One of the IL-6 target genes encodes the feedback inhibitor, suppressor of cytokine signalling 3 (SOCS3). SOCS3 is a rapidly induced [7–9] and short-lived [10] inhibitor of the JAK kinases that ensures limited and transient IL-6 signal transduction [11, 12]. *SOCS3* mRNA and protein expression are tightly regulated. Besides proteasomal degradation of the SOCS3 protein [13], *SOCS3* mRNA expression is controlled by mRNA stability-regulating signals targeting the 3′ untranslated region of *SOCS3* mRNA [14] and miRNAs that directly target SOCS3 mRNA [15]. *SOCS3* promoter activity is silenced by hypermethylation [16, 17]. Dysregulated expression of SOCS3 resulting in impaired inhibition of inflammatory responses is associated with uncontrolled inflammation and cancer [18]. We showed previously that glucocorticoids inhibit *SOCS3* expression and thus increase IL-6-induced STAT3 activation and acute-phase protein expression [19], providing a molecular mechanism of stress-induced regulation of inflammatory responses.

Other pathways involved in stress signalling also interfere with JAK/STAT signalling. As part of the cell’s antiviral response, the stress kinase, Protein Kinase RNA-activated (PKR) is canonically activated by binding double-stranded RNA generated upon virus infection [20] and subsequent auto-phosphorylation [21]. Activated PKR inhibits translation initiation by phosphorylating the eukaryotic translation initiation factor eIF2α chain (eIF2α) [22, 23]. Thus, PKR-dependent phosphorylation of eIF2α is an integral part of an efficient strategy to inhibit the synthesis of viral proteins by blocking cellular translation. Indeed, activation of PKR and eIF2α phosphorylation are essential for the integrated cellular response to diverse stressors [24–26]. In line with this concept, phosphorylation of eIF2α is also essential for the ER-stress response [27].

Besides activation of PKR by viral double-stranded RNA, PKR can be activated strongly by intragenic double-helical structures encoded by cellular genes. These structures have been discovered within the (pre-)mRNAs coding for inflammatory cytokines such as IFN-γ [28, 29] and TNF-α [30, 31] and within the *α-*, *β-* and *γ-globin* genes [32]. These intragenic RNA activators control the translation [28, 29] or splicing [30–32] of these mRNAs, respectively, in dependence on eIF2α phosphorylation. PKR was also suggested to interfere directly with epidermal growth factor (EGF)-dependent JAK/STAT signalling by affecting the expression of SOCS3 isoforms [33]. Expression of two different SOCS3 isoforms is caused by two alternative translational start sites, separated by 30 nucleotides within *SOCS3* mRNA. Within the long isoform of SOCS3, lysine at position 6 is evolutionary conserved and serves as a potential ubiquitination site, rendering SOCS3 protein short-lived. Due to the lack of this residue in the short SOCS3 isoform, this isoform is more resistant to proteasomal degradation [33]. Expression of the short isoform was reported to be favoured in the presence of activated PKR [33].

Here, we analysed the function of the isoform-specific N-terminal peptide of SOCS3 and evaluated the inhibitory potential of both isoforms on IL-6-induced signalling. In addition, we studied protein stability of both SOCS3 isoforms in an experimental setup that precludes any influence of signal transduction on SOCS3 protein stability. Furthermore, we examined which elements within *SOCS3* mRNA affect the ratio of SOCS3 isoforms expressed and re-evaluated the impact of PKR on SOCS3 isoform expression.

## Methods

### Cloning

The DNA sequence coding for *SOCS3* pre-mRNA was amplified from pUC57 SOCS3 (GenScript, Piscataway, NJ, USA) with the primers fw: 5′-TATCTGGGTACCGGATCCGCGGCTCCGACTTGGA-3′; rv: 5′-GTCGGCTCTAGAGTTTTTCATTAA-3′ (Thermo Fisher Scientific, Waltham, MA, USA) and cloned into pcDNA3 (Thermo Fisher Scientific) using BamHI and XbaI (Cell Signalling Technology (CST), Frankfurt/Main, Germany). pcDNA3-[*SOCS3* mRNA] was prepared by deletion of the intron within pcDNA3-[*SOCS3* pre-mRNA]. The DNA sequence coding for *SOCS3* mRNA was amplified from pcDNA3-[*SOCS3* pre-mRNA] using the primers fw: 5′-CCCCCCGGGATGCGGTAGCG-3′; rv: 5′-CTGGTCCCGAATCGAAGTCTCCG-3′ (Thermo Fisher Scientific). Phosphorylation and ligation of PCR products was done with T4 kinase (Thermo Fisher Scientific) and T4 ligase (Thermo Fisher Scientific) respectively. Site directed mutagenesis of pcDNA3-[*SOCS3* mRNA] to pcDNA3-[*SOCS3* cds dAUG^1^] was done with the primers fw: 5′-GTGCGCCGTGGTCACCCACAG-3′; rv: 5′-CTGTGGGTGACCACGGCGCAC-3′ (Thermo Fisher Scientific) to substitute GUG for the first AUG start codon. pcDNA3-[*SOCS3* cds], pcDNA3-[*SOCS3* cds dN] and pcDNA3-[*SOCS3* cds M12V] were kindly provided by A. Yoshimura (Keio University, Tokyo, Japan). pcDNA3-[*SOCS3* cds] codes for the full length coding sequence of the long SOCS3 isoform. pcDNA3-[*SOCS3* cds dN] lacks the 28 5′-nucleotides of the full length coding sequence. This construct thus codes for the SOCS3 short isoform as translation starts with the 2nd AUG. pcDNA3-[*SOCS3* cds M12V] codes for the full length coding sequence of the long SOCS3 isoform but the 2nd AUG codon has been replaced by a valine-coding codon GUG.

*SOCS3* mRNA d5’UTR was amplified from pcDNA3-[*SOCS3* pre-mRNA] using the primers fw: 5′-TATCTGAAGCTTTGCGCCATGGTCACCCACAGC-3′; rv: 5′-GTCGGCTCTAGAGTTTTTCATTAA-3′ (Thermo Fisher Scientific) to eliminate the *SOCS3* 5′-UTR. The PCR product was cloned into pcDNA3 using HindIII and XbaI (CST) to prepare pcDNA3-[*SOCS3* mRNA d5’UTR]. *SOCS3* mRNA d5’UTR dKozak was amplified from pcDNA3-[*SOCS3* pre-mRNA] using the primers fw: 5′-TATCTGAAGCTTATGGTCACCCACAGCA-3′; rv: 5′-GTCGGCTCTAGAGTTTTTCATTAA-3′ (Thermo Fisher Scientific) and cloned into pcDNA3 using HindIII and XbaI (CST) to prepare pcDNA3-[*SOCS3* mRNA d5’UTR dKozak].

*SOCS3* coding sequence was amplified from pcDNA3-[SOCS3 cds] using the primers fw: 5′-TATCTGAAGCTTATGGTCACCCACAGCA-3′; rv: 5′-GTCGGCGATATCTGCCCTTTGCGCCCTTTA-3′ (Thermo Fisher Scientific) and cloned into pcDNA5/FRT using HindIII and EcoRV (CST) to prepare pcDNA5/FRT-[*SOCS3* cds]. The DNA sequence coding for *SOCS3* cds dN was amplified from pcDNA3-[*SOCS3* cds dN] using the primers fw: 5′-TATCTGAAGCTTATGAGCCGCCCCCTGGACA-3′; rv: 5′-GTCGGCGATATCTGCCCTTTGCG CCCTTTA-3′ (Thermo Fisher Scientific) and cloned into pcDNA5/FRT using HindIII and EcoRV (CST) to prepare pcDNA5/FRT-[*SOCS3* cds dN]. The DNA sequence coding for *SOCS3* cds M12V was amplified from pcDNA3-[*SOCS3* cds M12V] using the primers fw: 5′-TATCTGAAGCTTATGGTCACCCACAGCA-3′; rv: 5′-GTCGGCGATATCTGC CCTTTGCGCCC TTTA-3′ (Thermo Fisher Scientific) and cloned into pcDNA5/FRT using HindIII and EcoRV (CST) to prepare pcDNA5/FRT-[*SOCS3* cds M12V]. The DNA sequence coding for *SOCS3* pre-mRNA was amplified from pcDNA3-[*SOCS3* pre-mRNA] using the primers fw: 5′-TATCTGAAGCTTGCGGCTCCGACTTGGA-3′; rv: 5′-ATGAGATATCTCTAGAGTTTTTCATTAA-3′ (Thermo Fisher Scientific) and cloned into pcDNA5/FRT using HindIII and EcoRV (CST) to prepare pcDNA5/FRT-[*SOCS3* pre-mRNA].

We were very cautious not to affect the Kozak sequences within the described constructs. The sequence surrounding the first AUG does not differ between *SOCS3* pre-mRNA, SOCS3 mRNA, *SOCS3* mRNA d5’UTR, *SOCS3* cds and *SOCS3* cds M12V. The sequence surrounding the first AUG is altered (or missing) in *SOCS3* mRNA d5’UTR dKozak, *SOCS3* cds dN, and *SOCS3* cds dAUG1. The sequence surrounding the 2nd AUG is not altered in *SOCS3* pre-mRNA, *SOCS3* mRNA, *SOCS3* mRNA d5’UTR, *SOCS3* mRNA d5’UTR dKozak, *SOCS3* cds, *SOCS3* cds dN, and *SOCS3* cds dAUG1. However, the sequence is altered in *SOCS3* cds M12V due to substitution of GUG to AUG (Additional file 1: Figure S1).

### Cell culture

Human embryonic kidney (HEK) 293 cells and HeLa cells (both DSMZ, Braunschweig, Germany) were grown in Dulbecco’s modified Eagles medium (DMEM; Thermo Fisher Scientific) supplemented with 10% FCS, streptomycin (100 U/mL), penicillin (100 μg/mL) at 37 °C in a water saturated atmosphere containing 5% CO_2_. HEK293 Flp-In™ T-Rex™ (HEK293 Flp-In, Thermo Fisher Scientific) cells were used to prepare doxycycline-inducible stable transfectants. Stable transfectants expressing either the long or short SOCS3 isoform in response to doxycycline (HEK293 Flp-In SOCS3 cds M12V cells or HEK293 Flp-In SOCS3 cds dN cells) were generated by liposomal transfection with pcDNA5/FRT-[*SOCS3* cds M12V] or pcDNA5/FRT-[*SOCS3* cds dN] following manufacturer’s instructions. HEK293 Flp-In SOCS3 cds M12V cells or HEK293 Flp-In SOCS3 cds dN cells were cultivated in DMEM supplemented with FCS (10 %), streptomycin (100 U/mL), penicillin (100 µg/mL), blasticidin (10 µg/mL) and hygromycin (50 µg/mL). Prior to stimulation, HEK293 or HeLa cells were starved for four hours. HepG2 cells (DSMZ, Braunschweig, Germany) were grown in DMEM + F12 (Thermo Fisher Scientific) supplemented with 10% FCS, streptomycin (100 U/mL) and penicillin (100 μg/mL) at 37 °C in a water saturated atmosphere containing 5% CO_2_. Prior to stimulation, HepG2 cells were starved overnight in medium without FCS. Cells were treated with hyper-IL-6 (hyIL-6, Conaris, Kiel, Germany), IL-6 (Conaris), doxycycline (Sigma-Aldrich, St. Louis, MO, USA), MG-132 (10 μM, Santa Cruz Biotechnology, Dallas, TX, USA), cycloheximide (50 μg/mL, Sigma Aldrich, St. Louis, MO, USA), poly I:C (InvivoGen, Toulouse, France), or salubrinal (12.5 μM, Santa Cruz Biotechnology) as indicated.

### Generation of PKR-deficient cells

HEK293 Flp-In™ T-Rex™ cells, HeLa cells or HepG2 cells were transfected with PKR CRISPR/Cas9 KO plasmids (sc-400,177) together with PKR HDR plasmids (sc-400,177-HDR) (Santa Cruz Biotechnology). Transfection was done using Lipofectamine 2000 (Thermo Fisher Scientific) according to manufacturer’s instructions. Puromycin-resistant cells were isolated and cultivated in DMEM (Thermo Fisher Scientific), supplemented with 10% FCS, streptomycin (100 U/mL), penicillin (100 μg/mL) and puromycin (2 μg/mL).

### Transfection

Liposomal transfection was performed in OptiMEM (Thermo Fisher Scientific) using Lipofectamine 2000 (Thermo Fisher Scientific) according to manufacturer’s instructions. Four hours after transfection, OptiMEM was replaced by DMEM supplemented with 10% FCS. Cells were harvested 24 h after transfection.

### Reporter gene assay (HEK cells)

300,000 HEK Flp-In SOCS3 cds dAUG^1^, SOCS3 cds M12V or SOCS3 cds dN cells were grown in six-well plates for 24 h. Transient transfection of 160 ng SOCS3 promoter luciferase reporter vector (pGL3-*SOCS3* LucPm (− 511/+ 929) (Shlomo Melmed, Los Angeles, CA, USA) and 160 ng β-galactosidase expression vector (pCR3 lacZ) per well was performed in OptiMEM using Lipofectamine 2000 according to manufacturer’s instructions (Thermo Fisher Scientific). Four hours after transfection, OptiMEM was replaced by DMEM supplemented with 10% FCS. 24 h after transfection cells were left untreated or treated with indicated amounts of doxycycline to induce varying expression levels of the long or short isoform of SOCS3, respectively. In addition, cells were stimulated with or without hyIL-6 (20 ng/mL) for 16 h. Cell lysis and luciferase assays were carried out using the luciferase assay system (Promega, Mannheim, Germany) according to manufacturer’s instructions. Activity of luciferase was normalized to β-galactosidase activity. Maximal promoter activity was set to 100%.

### Reporter gene assay (HeLa cells)

Three hundred fifty thousand HeLa cells were grown in six-well plates for 24 h. Transient transfection of 160 ng SOCS3 promoter luciferase reporter vector (pGL3-*SOCS3* LucPm (− 511/+ 929)) (Shlomo Melmed) and 160 ng β-galactosidase expression vector (pCR3 lacZ) per well was performed in OptiMEM using Lipofectamine 2000 according to manufacturer’s instructions (Thermo Fisher Scientific). In addition to this, cells were cotransfected with expression vectors for SOCS3 cds M12V or SOCS3 cds dN or empty vector as indicated to induce varying expression levels of the long or short isoform of SOCS3, respectively. For transfection of equimolar amounts of DNA (500 ng) the indicated amounts of the transfected expression vectors encoding SOCS3 cds M12V or SOCS3 cds dN were transfected along with empty vector. Four hours after transfection, OptiMEM was replaced by DMEM and cells were stimulated with or without hyIL-6 (10 ng/mL) for 16 h. Cell lysis and luciferase assays were carried out using the luciferase assay system (Promega, Mannheim, Germany) according to manufacturer’s instructions. Activity of luciferase was normalized to β-galactosidase activity. Maximal promoter activity was set to 100%.

### Quantitative RT PCR

Total RNA was isolated using the RNeasy Kit (Qiagen, Hilden, Germany) according to manufacturer’s instructions. 500 ng of total RNA was reverse transcribed into cDNA with Omniscript (Qiagen) using random hexameric primers (Carl Roth, Karlsruhe, Germany) according to manufacturer’s instructions. Real-time PCR was done on a Rotor-Gene Q (Qiagen) using SYBR Green PCR reagents (Thermo Fisher Scientific). Amplification of human SOCS3 cDNA and HPRT1 cDNA was achieved with specific primers for SOCS3 (fw: 5′-GGAGTTCCTGGACCAGTACG-3′, rv: 5′-ACATGGCACAAGCACAAGAA-3′) and HPRT1 (fw: 5′-TGACACTGGCAAAACAATGCA-3′, rv: 5′-GGTCCTTTTCACCAGCAAGCT-3′). The PCR reaction was performed in a final volume of 20 μl containing 2 μl cDNA, 250 nmol/l of each primer,10 μl SYBR green PCR buffer (Thermo Fisher Scientific), and 7 μl water. Following a 10 min denaturing step at 95 °C, amplification was performed in 40 cycles (10 s at 95 °C, 20 s at 60 °C, 30 s at 72 °C). *SOCS3* and *HPRT1* cDNAs were amplified in separate reaction wells in duplicates. The real-time PCR efficiencies were determined for each primer set from standard curves generated from serial dilutions of a cDNA sample of hyIL-6-stimulated (60 min, 10 ng/mL) HEK293 cells. Quantification of gene expression was calculated as described by Pfaffl et al. [34]. Maximal fold increase was set to 100%.

### Western blotting

For the isolation of cellular proteins cells were lysed in RIPA lysis buffer (50 mM Tris-HCl, pH 7.4, 150 mM NaCl, 0.5% NP-40, 15% Glycerol, supplemented with 10 μg/mL of each aprotinin, leupeptin and pepstatin as well as 0.8 μM Pefabloc (Roche, Mannheim, Germany), 1 mM NaF, and 1 mM Na_3_VO_4_). The protein concentration of the lysates was determined using Biorad Protein Assay following manufacturer’s instructions (Biorad, Munich, Germany). Proteins were separated by SDS-PAGE and transferred to a nitrocellulose membrane (0.2 μm, GE Healthcare, Chicago, IL, USA). For separation of the SOCS3 isoforms gels with a concentration of 12.5% polyacrylamide were used. Antigens were detected by incubation with specific primary antibodies (1:1000 in TBSN) followed by incubation with infrared-fluorescent-dye (IRDye®)-coupled secondary antibodies (1:10,000 in TBSN) (LI-COR Biosciences, Lincoln, NE, USA). Detection was done using the Odyssey Infrared Imaging System (LI-COR). List of primary antibodies: (p)S51 eIF2α (#3398), (p)Y705 STAT3 (#9145), STAT3 (#9139) (Cell Signalling Technology), eIF2α (sc-11,386), PKR (sc-707), (p)T451 PKR (sc-101,784) (Santa Cruz Biotechnology), SOCS3 (C204) (Immuno-Biological Laboratories, Fujioka, Japan) and α-Tubulin (Sigma-Aldrich Chemie, Munich, Germany). Western blots were quantified using Image Studio Software (LI-COR).

### Splicing assay

Total RNA was isolated using the RNeasy Kit (Qiagen) according to the manufacturer's instructions. After reverse transcription, amplification of cDNA coding *SOCS3* pre-mRNA and *SOCS3* mRNA was done by using the following primers: SOCS3 fw 5′-AAGGCTCCTTTGTGGACTT-3′, SOCS3 rv 5′-TCTTCCGACAGAGATGCT-3′ (Thermo Fisher Scientific) to give PCR products of 1300 bp or 800 bp, respectively. Amplification of GAPDH (GAPDH fw 5′-TGCCTCCTGCACCACCAACTGC-3′ and GAPDH rv 5′-AATGCCAGCCCCAGCGTCAAAG-3′ (Thermo Fisher Scientific)) served as an internal control. Amplification products were separated by agarose gel electrophoresis and stained with Roti-Stain (Carl Roth).

## Results

### IL-6 induces expression of two SOCS3 isoforms

**Fig. 1:**
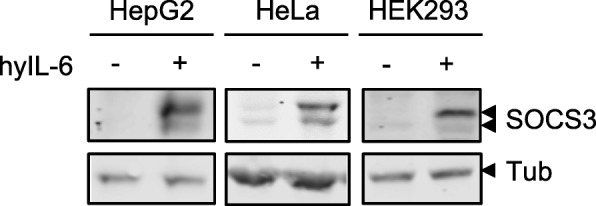
IL-6 induces the expression of two different isoforms of SOCS3. HepG2, HeLa, and HEK293 cells were stimulated with hyIL-6 (20 ng/mL) for 90 min or left untreated as indicated. SOCS3 and tubulin protein expression were evaluated by Western blotting. Arrowheads indicate the long and short isoforms of SOCS3. Representative results of *n* = 3 independent experiments are shown

The feedback inhibitor SOCS3 is rapidly induced in response to IL-6 and other IL-6-type cytokines [7–9]. However, differential expression of the two SOCS3 isoforms [33] and regulation of their expression in response to IL-6 has not been analysed in detail so far. To test whether IL-6 induces the expression of one or both SOCS3 isoforms, three unrelated human cell lines, hepatoma cells (HepG2), cervical cancer cells (HeLa), and embryonic kidney cells (HEK293), were stimulated with hyper-IL-6 (hyIL-6). Expression of both SOCS3 isoforms 90 min post stimulation with hyIL-6 was detected by Western blotting using an antibody that specifically detects the C-terminal region of both isoforms (Fig. 1). In all cell lines analysed, the long isoform was expressed more prominently than the short isoform. The short isoform is derived from an alternative translation initiation site located 33 nucleotides downstream from the first AUG start codon and thus shortened by 11 N-terminal amino acids [33]. Consequently, the molecular mass of the shorter isoform differs by 1 kDa from the longer isoform (Fig. 1). Thus, IL-6 induces endogenous expression of both SOCS3 protein isoforms in unrelated human cell lines. Of note, expression of the longer isoform exceeds that of the short isoform.

### The inhibitory potential of SOCS3 does not depend on isoform-specific N-terminal amino acids

**Fig. 2:**
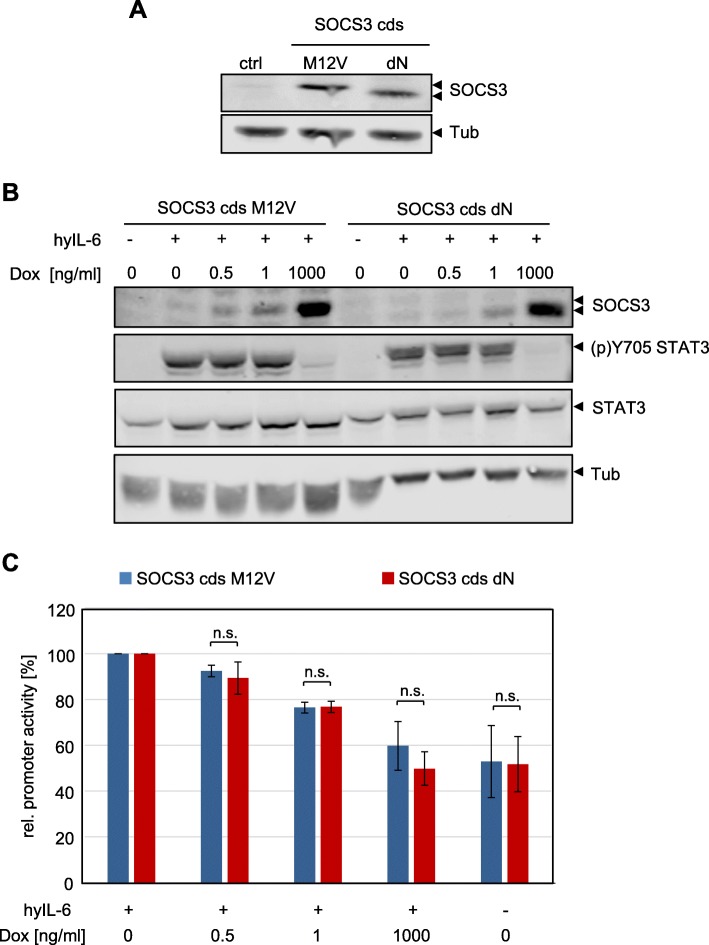
The inhibitory potential of SOCS3 does not depend on isoform-specific N-terminal peptide. **a** HEK293 cells were transfected with expression vectors for SOCS3 M12V or SOCS3 dN or empty vector (ctrl). Protein expression of SOCS3 isoforms and tubulin were monitored by Western blotting. Arrowheads indicate the long and short isoforms of SOCS3. **b** HEK293 FlpIn cells stably transfected to express doxycycline-dependently SOCS3 M12V or SOCS3 dN were treated for 16 h with the indicated amount of doxycycline to induce expression of SOCS3 isoforms and stimulated with hyIL-6 (20 ng/mL, 16 h) to induce IL-6-dependent STAT3-activation. STAT3 phosphorylation as well as STAT3, SOCS3 and tubulin protein expression were evaluated by Western blotting. Arrowheads indicate the long and short isoforms of SOCS3. Representative results of n = 3 independent experiments are shown. **c** Cells as in (B) were transfected with STAT3-reporter constructs and a β-galactosidase encoding expression vector. Cells were treated for 16 h with the indicated amount of doxycycline to induce SOCS3 isoform expression and with hyIL-6 (50 ng/mL, 16 h) to induce reporter gene expression. Luciferase activity was normalized to β-Galactosidase activity. Maximal reporter activity was set to 100%. Data are given as mean of three independent experiments ± SD. rANOVA: n.s. *p* > 0.05

We next asked whether the isoform-specific N-terminal part of the SOCS3 isoforms is needed for the inhibitory activity of SOCS3 during IL-6-induced JAK/STAT signalling. To study both isoforms independently, we expressed each isoform individually in HEK293 cells. To achieve exclusive expression of the long isoform, the downstream AUG codon (Met^12^) was replaced by a valine-coding codon, eliminating the second start codon (SOCS3 cds M12V). For exclusive expression of the short isoform, the coding region for the 11 N-terminal amino acids was deleted (SOCS3 cds dN). Transient transfection of HEK293 cells with vectors encoding the two isoforms resulted in exclusive expression of the respective isoform. Both constructs differ in molecular mass as expected (Fig. 2a).

To analyse the inhibitory potential of each isoform in a STAT3-dependent reporter assay, we established HEK293 FlpIn cells that express either SOCS3 cds M12V or SOCS3 cds dN in dependence on doxycycline (Fig. 2b). For this, HEK293 FlpIn cells expressing the long or the short SOCS3 isoform, respectively, were transfected with a STAT3-responsive reporter construct. Increasing doxycycline concentrations enhances SOCS3 protein expression as determined by Western blotting (Fig. 2b, top row). STAT3 phosphorylation after 16 h of hyIL-6 treatment is impaired at the highest SOCS3 expression (Fig. 2b, second and third rows). The activity of the reporter construct was determined after stimulation with hyIL-6 for 16 h and increasing amounts of doxycycline to induce the long or the short isoform of SOCS3, respectively (Fig. 2c). Reporter activation in the absence of doxycycline was set to 100%. Both SOCS3 isoforms inhibited similarly and dose-dependently the activation of the reporter construct at moderate SOCS3 expression levels (Fig. 2c). Excess of SOCS3 expression in response to 1 mg/mL doxycycline dampened the reporter activity to the level observed in the absence of any stimulation. To substantiate these observations, we tested the inhibitory power of SOCS3 isoforms in HeLa cells which are more responsive to IL-6 than HEK293 FlpIn cells. HeLa cells were transfected with the STAT3 reporter construct together with increasing amounts of expression vectors encoding the long or short SOCS3 isoform. Again, both SOCS3 isoforms inhibited similarly and dose-dependently the activation of the reporter constructs (Additional file 1: Figure S2), indicating that the specific N-terminal part of the long isoform of SOCS3 does not significantly influence the inhibitory activity of SOCS3.

### The short SOCS3 isoform is more stable than the long SOCS3 isoform

**Fig. 3:**
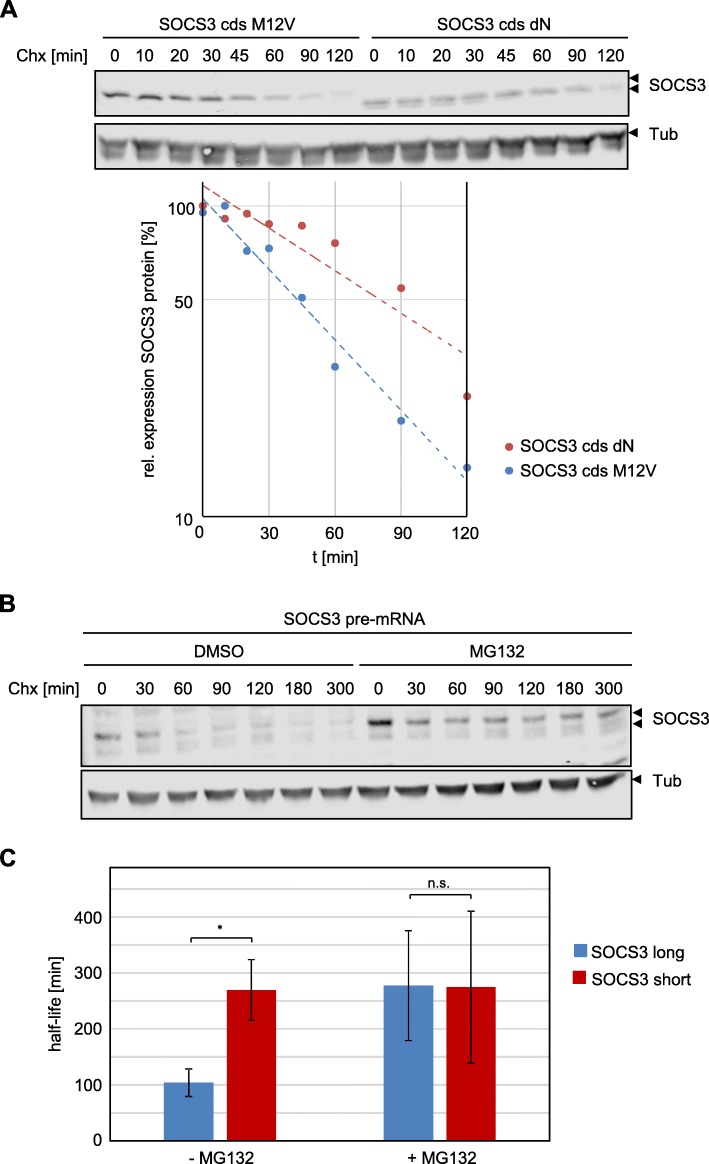
The short SOCS3 isoform is more stable than the long isoform of SOCS3. **a** HEK293 cells were transfected with expression vectors for SOCS3 M12 V or SOCS3 dN. Cells were treated with 50 μg/mL cycloheximide for the times indicated. SOCS3 and tubulin protein expression were evaluated by Western blotting. Arrowheads indicate the long and short isoforms of SOCS3. Lower panel: quantification of relative SOCS3 protein expression of the long (blue) and short SOCS3 (red) isoform. Expression was normalized to tubulin expression. Maximal SOCS3 protein expression was set to 100%. Representative results of *n* = 4 independent experiments are shown (Additional file 1: Figure S3). **b** HEK293 FlpIn cells stably transfected to express doxycycline-dependently *SOCS3* pre-mRNA were treated with 1 μg/mL doxycycline for 4 h to induce expression of the SOCS3 isoforms. After withdrawal of doxycycline, cells were treated with MG132 (10 μg/mL). After 15 min 50 μg/mL cycloheximide was added for the times indicated. SOCS3 and tubulin expression were evaluated by Western blotting. Arrowheads indicate the long and short isoforms of SOCS3. Representative results of *n* = 3 independent experiments are shown. For quantification see Additional file 1: Figure S4. **c** Quantification of the half-lives of the long (blue) and short SOCS3 (red) isoform in the absence or presence of MG132 from experiments as shown in Fig. 3b. Expression was normalized to tubulin expression. Maximal SOCS3 protein expression was set to 100% for each specific data set. Calculation of the half-lives was performed by linear regression for each specific data set. Data are given as mean of three independent experiments ± SD. Half-lives of the long and short SOCS3 isoform differ significantly in the absence of MG132 treatment (rANOVA: * p = 0.016) but are equal in the presence of MG132 (rANOVA: n.s. *p* > 0.05)

SOCS3 protein is known to be very short-lived [10]. Previous studies on growth factor-induced SOCS3 expression suggested that the EGF-induced shorter isoform is more stable than the long isoform [33]. Here, we compared the stability of both isoforms independently of any stimulus and in the absence of the respective other isoform. To this end, we expressed each isoform exclusively and subsequently blocked translation by adding cycloheximide to the cells. The decay of both SOCS3 protein isoforms was then monitored for up to 120 min. The blot in Fig. 3a and corresponding quantitation suggest that the short SOCS3 isoform (SOCS3 cds dN) is more stable than the long isoform (SOCS3 cds M12V), independently of a stimulus (Additional file 1: Figure S3).

To substantiate these results and to test whether the two isoforms of SOCS3 influence each other, we generated HEK293 FlpIn cells that express complete *SOCS3* pre-mRNA in dependence on doxycycline (HEK FlpIn *SOCS3* pre-mRNA). The resulting mRNA encodes both SOCS3 isoforms. We induced expression of *SOCS3* with doxycycline and subsequently blocked translation by adding cycloheximide to monitor the decay of the SOCS3 isoforms (Fig. 3b). As already shown for expression of endogenous SOCS3 (Fig. 1a), the short isoform was less abundant than the long isoform (Fig. 3b, lanes 1 and 8). As in Fig. 3a, the short SOCS3 isoform was significantly more stable (4.5 h) than the long isoform (1.7 h) (Fig. 3b, lanes 1 to 7; Fig. 3c; Additional file 1: Figure S4). The half-life of the long isoform confirms the one previously determined by classical pulse-chase approaches [10]. Initial studies of the half-life of SOCS3 isoforms suggested differential proteasomal degradation of the two isoforms, as the short isoform lacks a prominent ubiquitination side [33]. To test this, we applied the proteasomal inhibitor MG132 prior to cycloheximide treatment. MG132 stabilized the long isoform of SOCS3, whereas it did not affect the half-life of the short isoform (Fig. 3b, lanes 8–14; Fig. 3c; Additional file 1: Figure S4). This confirms the involvement of the proteasome for the degradation of the long SOCS3 isoform.

With these experiments, we confirm the results of Sasaki et al. [33] and conclude that the long SOCS3 isoform is less stable than the short isoform, despite the fact that expression of the long isoform is more pronounced than that of the short isoform.

### The UTRs and the first AUG codon in the *SOCS3* mRNA influence the ratio of SOCS3 isoform expression

**Fig. 4:**
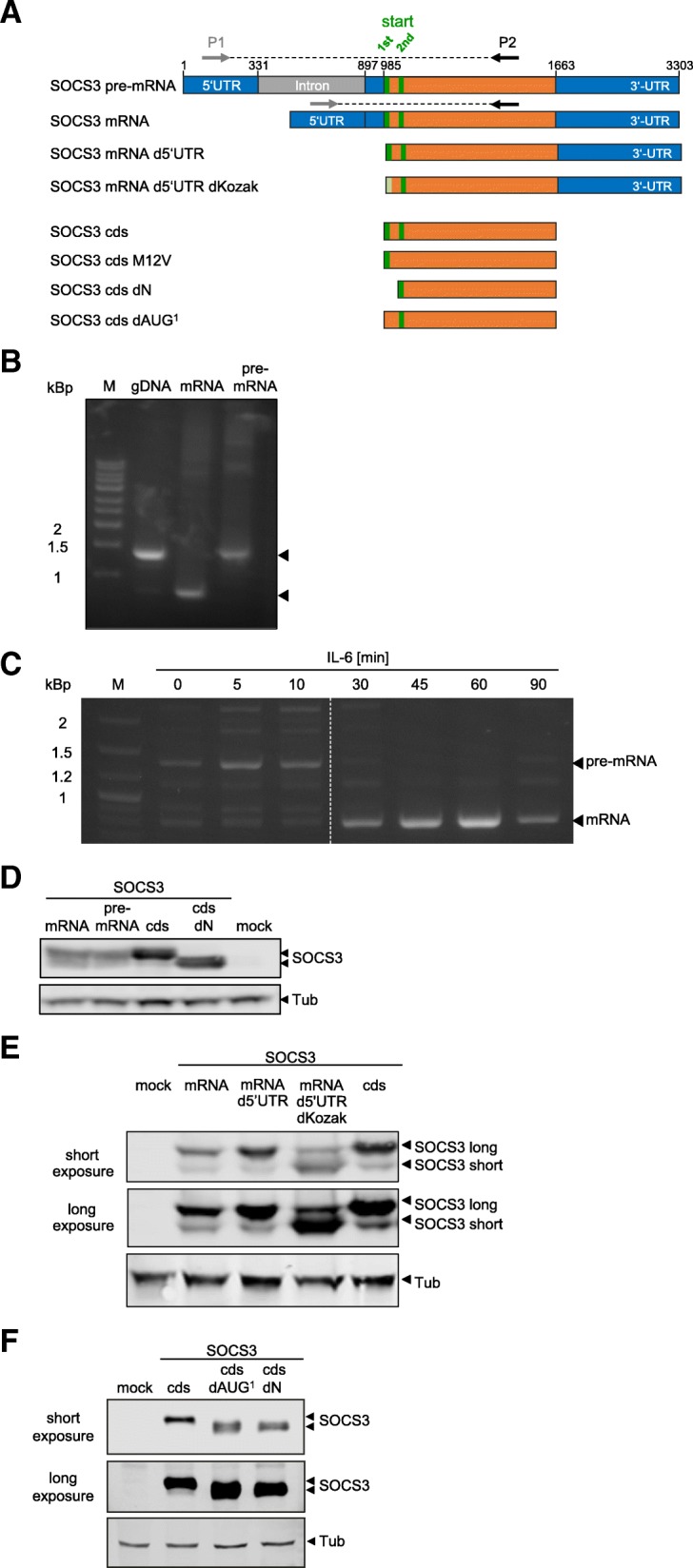
The UTRs and the first AUG codon influence the ratio of SOCS3 isoform expression. **a** Schema of the SOCS3 constructs analyzed in the following experiments (orange: coding sequence; grey: intron; blue: UTR; green: alternative translational start sites). P1 and P2 represent the primer pair used for amplification and discrimination of non-spliced *SOCS3* pre-mRNA and spliced *SOCS3* mRNA. For information on the Kozak sequence of the specific constructs see Additional file 1: Figure S1) (**b**) A specific primer pair corresponding to sequences located 5′ and 3′ to the single intron was used to discriminate spliced mRNA and un-spliced pre-mRNA (see Fig. 4a). For reference genomic DNA from untreated HepG2 cells was isolated and a DNA fragment corresponding to the unspliced pre-mRNA of *SOCS3* was amplified (lane 1). Expression vectors for *SOCS3* mRNA and *SOCS3* pre-mRNA (Fig. 4a) were used as templates for amplification of DNA fragments corresponding to *SOCS3* mRNA or unspliced *SOCS3* pre-mRNA, respectively (lanes 2 and 3). Amplification products were separated by agarose gel electrophoresis. **c** HepG2 cells were stimulated with 10 ng/mL IL-6 for the indicated times. mRNA was isolated, translated into cDNA and cDNA amplified as in Fig. 4b. Amplification products were separated on the same gel by gel electrophoresis. The dashed line indicates removed lanes. Exposure was identical to all parts of the gel. Amplicons corresponding to spliced mRNA and non-spliced pre-mRNA are indicated by arrows. For quantification see Additional file 1: Figure S5. **d** HEK293 cells were transfected with expression vectors for *SOCS3* pre-mRNA, *SOCS3* mRNA, the coding sequence of SOCS3 (SOCS3 cds), and the coding sequence of the short isoform of SOCS3 (SOCS3 dN) (Fig. 4a). SOCS3 and tubulin protein expression were evaluated by Western blotting. Arrowheads indicate the long and short isoforms of SOCS3. For quantification see Additional file 1: Figure S6. Representative results of *n* = 3 independent experiments are shown. For expression of the different SOCS3 mutants, we adjusted the amount of transfected DNA in accordance to vector size. **e** HEK293 cells were transfected with expression vectors for *SOCS3* mRNA, *SOCS3* mRNA d5’UTR, *SOCS3* mRNA d5’UTR dKozak, and SOCS3 cds. SOCS3 and tubulin protein expression was evaluated by Western blotting. Arrowheads indicate the long and short isoforms of SOCS3. For expression of the different SOCS3 mutants we adjusted the amount of transfected DNA in accordance to vector size. **f** HEK293 cells were transfected with expression vectors containing the coding sequence of SOCS3 (lane 2; cds), expression vectors where the first AUG in the coding sequence of the long SOCS3 isoform was mutated (SOCS3 dAUG^1^) or expression vectors were the coding sequence of the long isoform-specific peptide was deleted (SOCS3 dN). SOCS3 and tubulin protein expression were evaluated by Western blotting. Arrowheads indicate the long and short isoforms of SOCS3. Representative results of n = 3 independent experiments are shown

The *SOCS3* gene contains a single 566 bp intron within the 985 bp long 5′-UTR and a 1.6 kBp long 3′-UTR critical for regulating *SOCS3* mRNA half-life [14] (Fig. 4a). Whether *SOCS3* mRNA structure affects the ratio of SOCS3-isoform expression remains unknown. We first studied the potential impact of *SOCS3* mRNA structure on expression of the long and short SOCS3 isoform, respectively. To follow splicing of *SOCS3* pre-mRNA, a primer pair corresponding to sequences located 5′ and 3′ to the single intron was used to discriminate spliced mRNA from unspliced pre-mRNA. These primers were first used to amplify the corresponding gene region from genomic DNA (gDNA) of HepG2 cells and gave rise to a PCR product of about 1.4 kBp (Fig. 4b, lane 2). A PCR product of the same size was amplified from expression vector coding for complete *SOCS3* pre-mRNA (Fig. 4a and Fig. 4b, lane 4). An expression vector lacking the 566 bp intron (*SOCS3* mRNA; Fig. 4a) that encodes mature *SOCS3* mRNA gave rise to a PCR product of about 850 bp (Fig. 4b, lane 3). Next, we used this set of primers to monitor the kinetics of *SOCS3* pre-mRNA splicing in HepG2 cells stimulated with IL-6. Upon IL-6 stimulation, *SOCS3* pre-mRNA was induced rapidly and was spliced efficiently, with little, if any, pre-mRNA detectable 30 min post stimulation (Fig. 4c, Additional file 1: Figure S5).

Next, we asked whether the UTRs and *SOCS3* pre-mRNA splicing affect expression and SOCS3 isoform ratio. We compared the expression of long and short isoforms of SOCS3 encoded by vectors expressing complete *SOCS3* pre-mRNA, *SOCS3* mRNA, the coding sequence of SOCS3 (SOCS3 cds), or the coding sequence of the short isoform (SOCS3 cds dN) (Fig. 4a). All four vectors were transfected into HEK293 cells and expression of SOCS3 isoforms was detected by Western blot analysis (Fig. 4d). Transfection of the pre-mRNA and mRNA vectors resulted in expression of both SOCS3 isoforms. Expression of the long isoform was more pronounced than expression of the short isoform (Additional file 1: Figure S6), confirming results in Figs. 1 and 3b. This experiment indicates that the intron does not affect expression and ratio of the two isoforms (compare lanes 1 and 2 in Fig. 4d).

Deleting both 5′-UTR and 3′-UTRs from *SOCS3* mRNA strongly increased expression of the long protein isoform, whereas expression of the short isoform was not affected (compare lanes 2 and 3 in Fig. 4d). Apparently, the UTRs of *SOCS3* mRNA hinder utilization of the first AUG codon as translational start site yet do not affect use of the second AUG codon. To analyse the impact of the 5′-UTR on SOCS3 isoform expression, we expressed SOCS3 mRNA d5’UTR which lacks the 5′-UTR but retains the Kozak translational initiation consensus sequences. We monitored SOCS3 isoform expression by Western blotting (Fig. 4e). Expression of the long SOCS3 isoform by *SOCS3* mRNA d5'UTR was enhanced compared to those by *SOCS3* mRNA. This indicates that the 5′-UTR counteracts expression of the long SOCS3 isoform. Expression of the short isoform was not affected by the 5’-UTR. Furthermore, we compared SOCS3 isoform expression initiated from SOCS3 mRNA d5’UTR and SOCS3 cds to evaluate the impact of the 3′-UTR. The results shown in Fig. 4e suggest that the 3′-UTR does not affect SOCS3 isoform expression.

Additionally, we mutated the Kozak sequence surrounding the first AUG (*SOCS3* mRNA d5’UTR dKozak) to study the impact of the Kozak sequence on SOCS3 isoform expression. Expression of the SOCS3 long isoform was reduced and, interestingly, expression of the short SOCS3 isoform was enhanced. These results suggest that translational initiation from the 1st AUG hinders translational initiation from the 2nd AUG. This hypothesis is further supported by the results shown in Fig. 4f: deletion of the upstream sequence needed for synthesis of the long isoform increased the expression of the short isoform (compare lanes 2 and 4 in Fig. 4d), suggesting that use of the first AUG codon restricts usage of the second translation start codon. To examine this point in more detail, we mutated the first AUG in the coding sequence of the long SOCS3 isoform (*SOCS3* dAUG^1^). In cells transfected with this construct, only the short SOCS3 isoform was expressed (Fig. 4f, lane 3). Expression of the short isoform was strongly increased compared to that in cells transfected with an expression vector containing both AUGs (*SOCS3* cds). The expression was comparable to that in cells transfected with the coding sequence for the short isoform (*SOCS3* cds dN) (Fig. 4f). These results indicate that usage of the first AUG and not the presence of the long isoform-specific N-terminal nucleotides suppresses translational initiation from the second AUG.

We conclude that the 5′-UTR of *SOCS3* mRNA specifically downregulates expression of the long SOCS3 isoform and that expression of the long isoform suppresses synthesis of the short isoform.

### PKR knockout or activation of PKR does not affect expression of SOCS3 isoforms

The study of Sasaki et al. [33] suggested that activated PKR induces expression of the short isoform of SOCS3 by phosphorylating eIF2α. PKR, a serine/threonine protein kinase, is activated by binding of double-helical RNA that is either the product of viral replication or encoded in specifically folded inflammatory cytokine and *globin* mRNAs [29, 32].

**Fig. 5:**
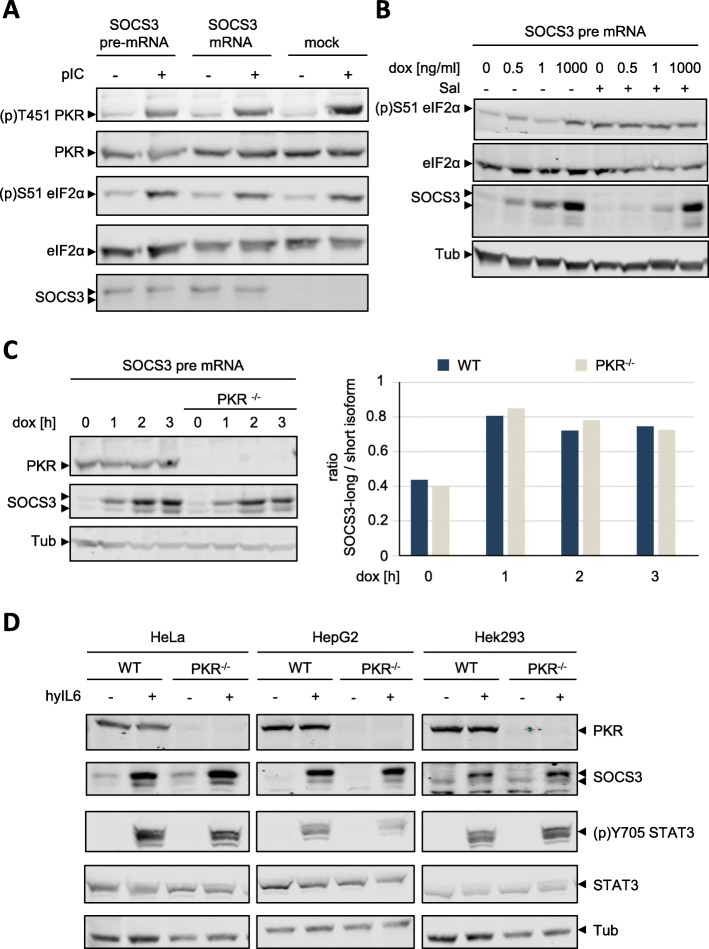
Neither PKR activation nor PKR knockout affect SOCS3 isoform expression. **a** HeLa cells were transfected with expression vectors encoding SOCS3 pre-mRNA or SOCS3 mRNA. After transfection cells were treated with 10 μg/mL pIC for 3 h. PKR phosphorylation ((p)T451), eIF2α phosphorylation ((p)S51) and PKR, eIF2α, SOCS3, and tubulin protein expression were evaluated by Western blotting. Arrowheads indicate the long and short isoform of SOCS3 respectively. Representative results of *n* = 3 independent experiments are shown. **b** HEK293 FlpIn SOCS3 pre-mRNA cells were treated with 12.5 μM salubrinal for 48 h and SOCS3 expression was induced by treatment with doxycycline with indicated concentrations for 3 h. eIF2α phosphorylation ((p)S51), eIF2α, SOCS3, and tubulin protein expression were evaluated by Western blotting. Arrowheads indicate the long and short isoforms of SOCS3. **c** PKR expression was eliminated in HEK293 FlpIn SOCS3 pre-mRNA cells using a CRISPR/Cas9 approach (PKR^−/−^). SOCS3 expression was induced by 1 μg/mL doxycycline for the times indicated. PKR, SOCS3, and tubulin protein expression were evaluated by Western blotting. Arrowheads indicate the long and short isoforms of SOCS3. Right panel: quantification of relative SOCS3 protein expression of the long and short SOCS3 isoform. Expression of the long isoform was normalized to the expression of the short isoform. Representative results of n = 3 independent experiments are shown. **d** PKR expression was eliminated in HepG2, Hek293, and HeLa cells using a CRISPR/Cas9 approach. Cells were stimulated with hyIL-6 (50 ng/mL) for 90 min or left untreated as indicated. PKR, SOCS3, (p)Y705 STAT3, STAT3, and tubulin protein expression were evaluated by Western blotting. Arrowheads indicate the long and short isoform of SOCS3. Representative results of *n* = 3 independent experiments are shown

We therefore tested whether activation of PKR with the pharmacological PKR activator and dsRNA mimetic, poly I:C, has an impact on the expression of SOCS3 isoforms. HeLa cells transfected with expression vectors for either *SOCS3* pre-mRNA or *SOCS3* mRNA to induce expression of both SOCS3 isoforms and control cells were treated with poly I:C. Poly I:C-induced threonine phosphorylation of PKR, serine phosphorylation of eIF2α and expression of SOCS3 isoforms were monitored by Western blotting. As shown in Fig. 5a, although poly I:C treatment resulted in PKR activation and phosphorylation of its eIF2α substrate, expression of SOCS3 isoforms was not altered upon activation of PKR.

We next tested whether the pharmacological inhibitor of eIF2α-dephosphorylation, salubrinal [27], affects SOCS3 isoform expression. SOCS3 expression was induced by increasing amounts of doxycycline in HEK293 FlpIn cells that express *SOCS3* pre-mRNA. Doxycycline induced dose dependent expression of both SOCS3 isoforms. Salubrinal increased eIF2α phosphorylation and slightly repressed SOCS3 protein expression, most probably by its impact on overall translation. However, it did not alter the ratio of the SOCS3 isoforms (Fig. 5b). To test whether the reduction of SOCS3 protein by salubrinal resulted from reduced translation of *SOCS3* mRNA, we analysed the effect of salubrinal on *SOCS3* mRNA expression. Indeed, salubrinal did not affect the extent of *SOCS3* mRNA expression (Additional file 1: Figure S7).

Using a CRISPR/Cas9 approach, we next generated PKR-deficient HEK293 FlpIn cells expressing *SOCS3* pre-mRNA to further elaborate the impact of PKR on SOCS3 isoform expression. Complete elimination of PKR expression also had no impact on the expression and ratio of the two SOCS3 isoforms (Fig. 5c). Furthermore, PKR knockout did not affect *SOCS3* mRNA expression (Additional file 1: Figure S8). These results do not support the concept that either activation of PKR or PKR-dependent phosphorylation of eIF2α affects SOCS3 isoform expression.

Finally, we evaluated the impact of PKR on IL-6-induced expression of endogenous SOCS3 isoforms. Using a CRISPR/Cas9 approach, we generated PKR-deficient HeLa, HepG2 and HEK293 cells. SOCS3 expression was induced by treating the cells with hyIL-6 for 90 min. As already shown in Fig. 1, hyIL-6 activated STAT3 and induced the expression of both SOCS3 isoforms in all three wild-type cell lines (Fig. 5d). Knockout of PKR expression affected neither hyIL-6-induced expression of SOCS3 isoforms nor hyIL-6-dependent STAT3 phosphorylation.

In summary, none of these different experimental approaches for testing the impact of the PKR pathway on SOCS3 expression supports the hypothesis that relative expression of SOCS3 isoforms is affected by PKR activation or eIF2α phosphorylation. By contrast, the UTRs and first AUG codon in *SOCS3* mRNA influence the ratio of SOCS3 isoform expression.

## Discussion

IL-6 is a central regulator of inflammation and thus highly relevant when the organism has to cope with infections or other traumata. Despite these beneficial functions of IL-6, dysregulated IL-6-induced signalling (e.g. hyper-activation of JAK/STAT signalling) contributes to the development of pathologies such as autoimmune diseases, chronic inflammation, or even cancer. Thus, guaranteeing fine-tuned signal transmission by IL-6 is of major importance for the organism. Several molecular mechanisms that regulate signal transmission through the IL-6 pathway have evolved. Most of these constitute negative feedback mechanisms such as cytokine-induced receptor internalization [35, 36], activation of cytoplasmic [37–39] or nuclear [40] phosphatases, and induction of kinase inhibitors of the SOCS family [7–9, 12].

Besides cell-intrinsic regulation, IL-6 signalling is also affected extrinsically by crosstalk with pro-inflammatory cytokines such as IL-1β and TNF-α and with stress stimuli [41, 42]. Thus, IL-1β impairs IL-6 signal transduction by inducing MK2-dependent phosphorylation and subsequent internalization and degradation of the signal-transducing receptor subunit gp130 [43]. Furthermore, NF-κB, activated in response to IL-1β, acts directly on IL-6-responsive promoter elements of acute-phase proteins [44, 45]. TNF-α increases *SOCS3* mRNA stability by a p38/MK2 stress-kinase-dependent mechanism and thus counteracts IL-6 signalling [14]. Glucocorticoids increase acute-phase protein expression in the liver [46, 47] by reducing IL-6-induced SOCS3 expression, likely through control of *SOCS3* mRNA translation [19].

The stress kinase PKR was suggested to affect SOCS3 expression [33]. PKR is activated by dsRNA generated upon viral infections [21, 22]. Activated PKR phosphorylates eukaryotic initiation factor eIF2α and thereby inhibits translation initiation [20, 23]. Likewise, eIF2α is phosphorylated by PERK in response to cellular stress induced by the unfolded protein response [24]. These examples underline the central role of eIF2α phosphorylation in limiting protein synthesis in stress situations. However, PKR is also activated by intragenic double-helical structures within (pre-)mRNAs encoding inflammatory cytokines. Short RNA elements within *IFN-γ* mRNA as well as within *TNF-α* and *globin* pre-mRNA control translation and splicing of these mRNAs, respectively, by strongly activating PKR [28–31]. Furthermore, PKR has been suggested to act on EGF-dependent JAK/STAT signalling by regulating the expression of the two SOCS3 isoforms. PKR was reported to promote expression of the short, more stable SOCS3 isoform [33]. However, it remained unclear whether EGF affects the half-life of the two isoforms, e.g., by inducing differential degradation of the isoforms or whether the stability of the two SOCS3 isoforms differs intrinsically. The decay of each isoform was not analysed in the absence of the other isoform to exclude mutual interaction. Little is known about relative expression of the two SOCS3 isoforms induced in response to other stimuli.

Here, we examined the role of PKR activation in IL-6 signalling and IL-6-dependent expression of the two SOCS3 protein isoforms. We demonstrate IL-6-induced expression of both isoforms in three independent human cell lines. Using a cytokine-independent cellular system that can be triggered to express exclusively either the long or the short isoform of SOCS3, we show that the short isoform is more stable than the long isoform, independent of cytokine or growth factor signalling and independent of the presence of the respective other isoform. These results indicate that the half-lives of the two SOCS3 isoforms are intrinsic parameters of the proteins, independent of stimulus.

Although the more stable short SOCS3 isoform might be expected to reach higher levels of expression than the less stable long isoform, expression of both isoforms from native mRNA yielded a more pronounced expression of the short-lived long isoform. This suggests that the steady-state ratio of the SOCS3 isoforms is not regulated primarily by protein stability. Previous studies showed that the 3′-UTR affects *SOCS3* mRNA stability [14]. In the present study, we show that the 5'-UTR and the first AUG codon in native *SOCS3* mRNA influence the ratio of SOCS3 isoform expression: The 5'-UTR of *SOCS3* mRNA limits expression of the long isoform but not of the short isoform. The 5′-UTR sequence hinders usage of the first AUG as translation start codon. We further hypothesised that translation from the second AUG is impeded within the native *SOCS3* mRNA. Indeed, upon elimination of the nucleotide sequence encoding the extra N-terminal amino acids of the long isoform, expression of the short isoform increased. This supports the concept that scanning ribosomes prefer the first AUG as translation start. This hypothesis was substantiated by mutation of the first AUG and mutation of the Kozak consensus sequence surrounding the first AUG. Both mutations resulted in specific and strong expression of the short SOCS3 isoform. Actually, the sequence surrounding the first start codon (TGCGCC**AUG**G) corresponds almost perfectly to the Kozak consensus sequence (GCCA/GCC**AUG**G) [48] whereas the sequence surrounding the second start codon is less (GCCGGG**AUG**A) optimal. Though preferential, translation starting from the first AUG codon is impaired by the upstream UTR. Strikingly, the N-terminal part of SOCS3 is highly conserved (Additional file 1: Figure S9). Both methionine^1^ and methionine^12^ but also lysine^6^, suggested to destabilise specifically the long isoform, are conserved in a wide range of species.

Our results thus show a tight regulation of the expression ratio of the unstable long SOCS3 isoform and the more stable short isoform. Alternative AUG start codons within a single transcript can contribute to diversity of the proteome, however, their functional significance remains controversial [49]. The organism may benefit from a more pronounced expression of the long SOCS3 isoform, as a short half-life of a regulatory protein provides dynamic regulation ensuring rapid response times. Conceivably, expression of this isoform could be regulated by proteins or non-coding RNAs binding to the 5′-UTR, as the 5′-UTR impedes usage of the first AUG start codon.

SOCS3 is known as kinase inhibitor and is also part of an E3 ligase complex. The latter function depends on the C-terminal SOCS-box [50]. So far, we have no evidence that the 11 N-terminal amino acids regulate the C-terminal SOCS-box. However, we cannot rule out the possibility that the SOCS3 isoforms specifically influence E3 ligase activity.

In contrast to the results of Sasaki et al. [33], activation of PKR by the double-stranded RNA mimic poly I:C did not affect exogenous expression of the two SOCS3 isoforms in the cellular systems analysed here. Also, stabilising eIF2α phosphorylation with salubrinal did not affect the ratio of SOCS3 isoform expression. Finally, knockout of the PKR gene did not change the relative expression of SOCS3 isoforms. These experiments do not support a role for PKR activation, PKR expression, or phosphorylation of eIF2α substrate on the ratio of SOCS3 isoform expression. This was further substantiated for IL-6-induced expression of endogenous SOCS3 protein in a broader set of cell lines lacking PKR expression. Sasaki et al. studied the effect of PKR on SOCS3 isoform expression by using SOCS3 cDNA lacking the 5’-UTR [33]. In clear contrast, we studied the effect of PKR on SOCS3 isoform expression by using SOCS3 pre-mRNA and SOCS3 mRNA containing both UTRs (Fig. 5). Thus, one might conclude that the presence of UTRs determines the function of PKR in SOCS3 isoform expression. Additionally, we cannot exclude that other stress-activated protein kinases substitute for PKR in our experiments. Furthermore, Sasaki et al. analysed SOCS3 isoform expression in response to IFNγ, IL-10, and EGF. We induced SOCS3 expression in response to IL-6-signalling or exogenously in the absence of any additional stimulus. Thus, one might speculate that the role of PKR depends on the specific stimulus. Interestingly, no obvious differences in the inhibitory potential of both SOCS3 isoforms were evident even when both isoforms were analysed separately. Thus, the inhibitory potential of SOCS3 does not depend on the isoform-specific N-terminal amino acids and the specific half-life of each isoform, but solely on the expression level of each SOCS3 isoform that depends on the UTRs and on the AUG start codon used.

## Conclusions

This study focused on the regulation of SOCS3 isoform expression. Both isoforms show a similar potential for inhibiting IL-6 signalling but differ in their half-lives. Expression of the isoforms depends on intragenic elements but is independent of PKR signalling. Our data add insight into the complex regulation of SOCS3 expression by extrinsic and intrinsic molecular mechanisms, relevant in light of the fact that SOCS3 expression is often dysregulated in inflammatory diseases.

## Additional file

Additional file 1: **Figure S1.** Kozak consensus sequences in the *SOCS3* constructs analysed. **Figure S2.** The inhibitory potential of SOCS3 in does not depend on isoform-specific N-terminal peptide. **Figure S3.** Half-lives of SOCS3 isoforms in HEK293 cells. **Figure S4.** Quantification of the SOCS3 degradation. **Figure S5.** Quantification of *SOCS3* pre-mRNA and *SOCS3* mRNA. **Figure S6.** Quantification of SOCS3 isoform expression. **Figure S7.** Salubrinal does not affect *SOCS3* mRNA expression. **Figure S8.** PKR knockout does not affect *SOCS3* mRNA expression. **Figure S9.** SOCS3 protein-sequence homology at the N-terminal end. (PDF 142 kb)

## Data Availability

The datasets used and/or analysed during the current study are available from the corresponding authors on reasonable request.

## Abbreviations

eIF2α: eukaryotic translation initiation factor 2 α-chain
IL: interleukin
JAK: Janus kinase
PKR: protein kinase RNA-activated
SOCS3: suppressor of cytokine signalling 3
STAT: signal transducer and activator of transcription
UTR: untranslated region

## References

[1] Baumann H, Jahreis GP, Morella KK (1990). Interaction of cytokine- and glucocorticoid-response elements of acute-phase plasma protein genes. Importance of glucocorticoid receptor level and cell type for regulation of the elements from rat alpha 1-acid glycoprotein and beta-fibrinogen genes. J Biol Chem.

[2] Baumann H, Richards C, Gauldie J (1987). Interaction among hepatocyte-stimulating factors, interleukin 1, and glucocorticoids for regulation of acute phase plasma proteins in human hepatoma (HepG2) cells. J Immunol.

[3] Andus T, Geiger T, Hirano T, Kishimoto T, Tran-Thi TA, Decker K, Heinrich PC (1988). Regulation of synthesis and secretion of major rat acute-phase proteins by recombinant human interleukin-6 (BSF-2/IL-6) in hepatocyte primary cultures. Eur J Biochem.

[4] Stahl N, Boulton TG, Farruggella T, Ip NY, Davis S, Witthuhn BA, Quelle FW, Silvennoinen O, Barbieri G, Pellegrini S (1994). Association and activation of Jak-Tyk kinases by CNTF-LIF-OSM-IL-6 β receptor components. Science.

[5] Stahl N, Farruggella TJ, Boulton TG, Zhong Z, Darnell JE, Yancopoulos GD (1995). Choice of STATs and other substrates specified by modular tyrosine-based motifs in cytokine receptors. Science.

[6] Lütticken C, Wegenka UM, Yuan J, Buschmann J, Schindler C, Ziemiecki A, Harpur AG, Wilks AF, Yasukawa K, Taga T (1994). Association of transcription factor APRF and protein kinase Jak 1 with the Interleukin-6 signal transducer gp 130. Science.

[7] Starr R, Willson TA, Viney EM, Murray LJ, Rayner JR, Jenkins BJ, Gonda TJ, Alexander WS, Metcalf D, Nicola NA, Hilton DJ (1997). A family of cytokine-inducible inhibitors of signalling. Nature.

[8] Endo TA, Masuhara M, Yokouchi M, Suzuki R, Sakamoto H, Mitsui K, Matsumoto A, Tanimura S, Ohtsubo M, Misawa H (1997). A new protein containing an SH2 domain that inhibits JAK kinases. Nature.

[9] Naka T, Narazaki M, Hirata M, Matsumoto T, Minamoto S, Aono A, Nishimoto N, Kajita T, Taga T, Yoshizaki K (1997). Structure and function of a new STAT-induced STAT inhibitor. Nature.

[10] Siewert E, Müller-Esterl W, Starr R, Heinrich PC, Schaper F (1999). Different protein turnover of interleukin-6-type cytokine signalling components. Eur J Biochem.

[11] Fischer P, Lehmann U, Sobota RM, Schmitz J, Niemand C, Linnemann S, Haan S, Behrmann I, Yoshimura A, Johnston JA (2004). The role of the inhibitors of interleukin-6 signal transduction SHP2 and SOCS3 for desensitization of interleukin-6 signalling. Biochem J.

[12] Yang XP, Schaper F, Teubner A, Lammert F, Heinrich PC, Matern S, Siewert E (2005). Interleukin-6 plays a crucial role in the hepatic expression of SOCS3 during acute inflammatory processes in vivo. J Hepatol.

[13] Williams JJ, Munro KM, Palmer TM (2014). Role of ubiquitylation in controlling suppressor of cytokine signalling 3 (SOCS3) function and expression. Cells.

[14] Ehlting C, Lai WS, Schaper F, Brenndorfer ED, Matthes RJ, Heinrich PC, Ludwig S, Blackshear PJ, Gaestel M, Häussinger D, Bode JG (2007). Regulation of suppressor of cytokine signaling 3 (SOCS3) mRNA stability by TNF-alpha involves activation of the MKK6/p38MAPK/MK2 cascade. J Immunol.

[15] Li Y, Luo T, Wang L, Wu J, Guo S (2016). MicroRNA-19a-3p enhances the proliferation and insulin secretion, while it inhibits the apoptosis of pancreatic beta cells via the inhibition of SOCS3. Int J Mol Med.

[16] Kim MH, Kim MS, Kim W, Kang MA, Cacalano NA, Kang SB, Shin YJ, Jeong JH (2015). Suppressor of cytokine signaling (SOCS) genes are silenced by DNA hypermethylation and histone deacetylation and regulate response to radiotherapy in cervical cancer cells. PLoS One.

[17] He B, You L, Uematsu K, Zang K, Xu Z, Lee AY, Costello JF, McCormick F, Jablons DM (2003). SOCS-3 is frequently silenced by hypermethylation and suppresses cell growth in human lung cancer. Proc Natl Acad Sci U S A.

[18] Inagaki-Ohara Kyoko, Kondo Taisuke, Ito Minako, Yoshimura Akihiko (2013). SOCS, inflammation, and cancer. JAK-STAT.

[19] Dittrich A, Khouri C, Sackett SD, Ehlting C, Böhmer O, Albrecht U, Bode JG, Trautwein C, Schaper F (2012). Glucocorticoids increase interleukin-6-dependent gene induction by interfering with the expression of the suppressor of cytokine signaling 3 feedback inhibitor. Hepatology.

[20] Nallagatla SR, Toroney R, Bevilacqua PC (2011). Regulation of innate immunity through RNA structure and the protein kinase PKR. Curr Opin Struct Biol.

[21] Zhang F, Romano PR, Nagamura-Inoue T, Tian B, Dever TE, Mathews MB, Ozato K, Hinnebusch AG (2001). Binding of double-stranded RNA to protein kinase PKR is required for dimerization and promotes critical autophosphorylation events in the activation loop. J Biol Chem.

[22] Kaempfer R, Kaufman J (1973). Inhibition of cellular protein synthesis by double-stranded RNA: inactivation of an initiation factor. Proc Natl Acad Sci U S A.

[23] Sonenberg N, Hinnebusch AG (2009). Regulation of translation initiation in eukaryotes: mechanisms and biological targets. Cell.

[24] Harding HP, Zhang Y, Zeng H, Novoa I, Lu PD, Calfon M, Sadri N, Yun C, Popko B, Paules R (2003). An integrated stress response regulates amino acid metabolism and resistance to oxidative stress. Mol Cell.

[25] Muaddi H, Majumder M, Peidis P, Papadakis AI, Holcik M, Scheuner D, Kaufman RJ, Hatzoglou M, Koromilas AE (2010). Phosphorylation of eIF2alpha at serine 51 is an important determinant of cell survival and adaptation to glucose deficiency. Mol Biol Cell.

[26] Tsaytler P, Harding HP, Ron D, Bertolotti A (2011). Selective inhibition of a regulatory subunit of protein phosphatase 1 restores proteostasis. Science.

[27] Boyce M, Bryant KF, Jousse C, Long K, Harding HP, Scheuner D, Kaufman RJ, Ma D, Coen DM, Ron D, Yuan J (2005). A selective inhibitor of eIF2alpha dephosphorylation protects cells from ER stress. Science.

[28] Ben-Asouli Y, Banai Y, Pel-Or Y, Shir A, Kaempfer R (2002). Human interferon-gamma mRNA autoregulates its translation through a pseudoknot that activates the interferon-inducible protein kinase PKR. Cell.

[29] Cohen-Chalamish S, Hasson A, Weinberg D, Namer LS, Banai Y, Osman F, Kaempfer R (2009). Dynamic refolding of IFN-gamma mRNA enables it to function as PKR activator and translation template. Nat Chem Biol.

[30] Namer LS, Osman F, Banai Y, Masquida B, Jung R, Kaempfer R (2017). An ancient pseudoknot in TNF-α pre-mRNA activates PKR, inducing eIF2α phosphorylation that potently enhances splicing. Cell Rep.

[31] Osman F, Jarrous N, Ben-Asouli Y, Kaempfer R (1999). A cis-acting element in the 3′-untranslated region of human TNF-alpha mRNA renders splicing dependent on the activation of protein kinase PKR. Genes Dev.

[32] Ilan L, Osman F, Namer LS, Eliahu E, Cohen-Chalamish S, Ben-Asouli Y, Banai Y, Kaempfer R (2017). PKR activation and eIF2alpha phosphorylation mediate human globin mRNA splicing at spliceosome assembly. Cell Res.

[33] Sasaki A, Inagaki-Ohara K, Yoshida T, Yamanaka A, Sasaki M, Yasukawa H, Koromilas AE, Yoshimura A (2003). The N-terminal truncated isoform of SOCS3 translated from an alternative initiation AUG codon under stress conditions is stable due to the lack of a major ubiquitination site, Lys-6. J Biol Chem.

[34] Pfaffl MW (2001). A new mathematical model for relative quantification in real-time RT-PCR. Nucleic Acids Res.

[35] Graeve L, Korolenko TA, Hemmann U, Weiergraber O, Dittrich E, Heinrich PC (1996). A complex of the soluble interleukin-6 receptor and interleukin-6 is internalized via the signal transducer gp130. FEBS Lett.

[36] Zohlnhöfer D, Graeve L, Rose-John S, Schooltink H, Dittrich E, Heinrich PC (1992). The hepatic interleukin-6 receptor. Down-regulation of the interleukin-6 binding subunit (gp80) by its ligand. FEBS Lett.

[37] Kim HK, Hawley TS, Hawley RG, Baumann H (1998). Protein tyrosine phosphatase 2 (SHP-2) moderates signaling by gp130 but is not required for the induction of acute-phase plasma protein genes in hepatic cells. Mol Cell Biol.

[38] Lehmann U, Schmitz J, Weissenbach M, Sobota RM, Hörtner M, Friederichs K, Behrmann I, Tsiaris W, Sasaki A, Schneider-Mergener J (2003). SHP2 and SOCS3 contribute to Tyr-759-dependent attenuation of interleukin-6 signaling through gp130. J Biol Chem.

[39] Schaper F, Gendo C, Eck M, Schmitz J, Grimm C, Anhuf D, Kerr IM, Heinrich PC (1998). Activation of the protein tyrosine phophatase SHP2 via the interleukin-6 signal transducing receptor protein gp130 requires JAK1 and limits acute-phase protein expression. Biochem J.

[40] Yamamoto T, Sekine Y, Kashima K, Kubota A, Sato N, Aoki N, Matsuda T (2002). The nuclear isoform of protein-tyrosine phosphatase TC-PTP regulates interleukin-6-mediated signaling pathway through STAT3 dephosphorylation. Biochem Biophys Res Commun.

[41] Ahmed ST, Ivashkiv LB (2000). Inhibition of IL-6 and IL-10 signaling and Stat activation by inflammatory and stress pathways. J Immunol.

[42] Ahmed ST, Mayer A, Ji JD, Ivashkiv LB (2002). Inhibition of IL-6 signaling by a p38-dependent pathway occurs in the absence of new protein synthesis. J Leukoc Biol.

[43] Radtke S, Wüller S, Yang XP, Lippok BE, Mütze B, Mais C, Schmitz-van de Leur HS, Bode JG, Gaestel M, Heinrich PC (2010). Cross-regulation of cytokine signalling: pro-inflammatory cytokines restrict IL-6 signalling through receptor internalisation and degradation. J Cell Sci.

[44] Yang XP, Albrecht U, Zakowski V, Sobota RM, Häussinger D, Heinrich PC, Ludwig S, Bode JG, Schaper F (2004). Dual function of interleukin-1β for the regulation of interleukin-6-induced suppressor of cytokine signaling 3 expression. J Biol Chem.

[45] Albrecht U, Yang X, Asselta R, Keitel V, Tenchini ML, Ludwig S, Heinrich PC, Häussinger D, Schaper F, Bode JG (2007). Activation of NF-kappaB by IL-1beta blocks IL-6-induced sustained STAT3 activation and STAT3-dependent gene expression of the human gamma-fibrinogen gene. Cell Signal.

[46] Rogatsky I, Ivashkiv LB (2006). Glucocorticoid modulation of cytokine signaling. Tissue Antigens.

[47] Paul C, Seiliez I, Thissen JP, Le Cam A (2000). Regulation of expression of the rat SOCS-3 gene in hepatocytes by growth hormone, interleukin-6 and glucocorticoids mRNA analysis and promoter characterization. Eur J Biochem.

[48] Kozak M (2005). Regulation of translation via mRNA structure in prokaryotes and eukaryotes. Gene.

[49] Bazykin GA, Kochetov AV (2011). Alternative translation start sites are conserved in eukaryotic genomes. Nucleic Acids Res.

[50] Babon JJ, Sabo JK, Zhang JG, Nicola NA, Norton RS (2009). The SOCS box encodes a hierarchy of affinities for Cullin5: implications for ubiquitin ligase formation and cytokine signalling suppression. J Mol Biol.

