# Supplementary material for: Intragenic regulation of SOCS3 isoforms
Source: Cell Commun Signal. 2019 Jun 25;17:70. doi: 10.1186/s12964-019-0379-6 (PMC6593527; doi:10.1186/s12964-019-0379-6)
Supplement: Supplementary file 1 — Figure S1. Kozak consensus sequences in the SOCS3 constructs analysed. Figure S2. The inhibitory potential of SOCS3 in does not depend on isoform-specific N-terminal peptide. Figure S3. Half-lives of SOCS3 isoforms in HEK293 cells. Figure S4. Quantification of the SOCS3 degradation. Figure S5. Quantification of SOCS3 pre-mRNA and SOCS3 mRNA. Figure S6. Quantification of SOCS3 isoform expression. Figure S7. Salubrinal does not affect SOCS3 mRNA expression. Figure S8. PKR knockout does not affect SOCS3 mRNA expression. Figure S9. SOCS3 protein-sequence homology at the N-terminal end. (PDF 142 kb) [file 12964_2019_379_MOESM1_ESM.pdf]

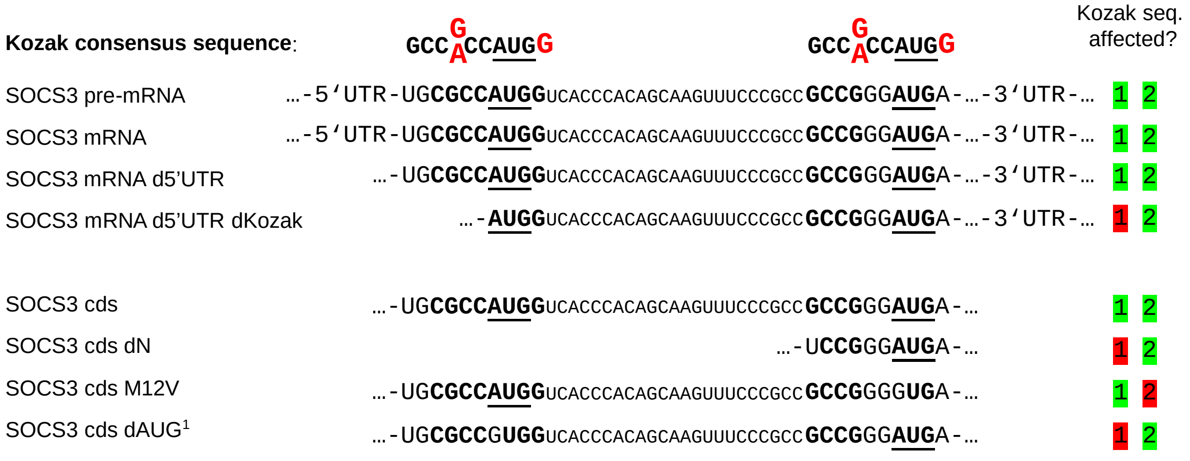

Supplementary Fig. S1. Kozak consensus sequences in the *SOCS3* constructs analysed

Alignment of the Kozak consensus sequence with the nucleotides surrounding the first and second translation start codon within the SOCS3 RNA sequences encoded by the different constructs used in this study. First and second translation initiation codon are underlined. Nucleotides matching the Kozak consensus sequence are indicated by bold letters. Right column indicates whether the relevant translation initiation site is affected compared to the wild-type pre-mRNA sequence (SOCS3 pre-mRNA): complete alignment is indicated in green whereas significant alterations are indicated in red.

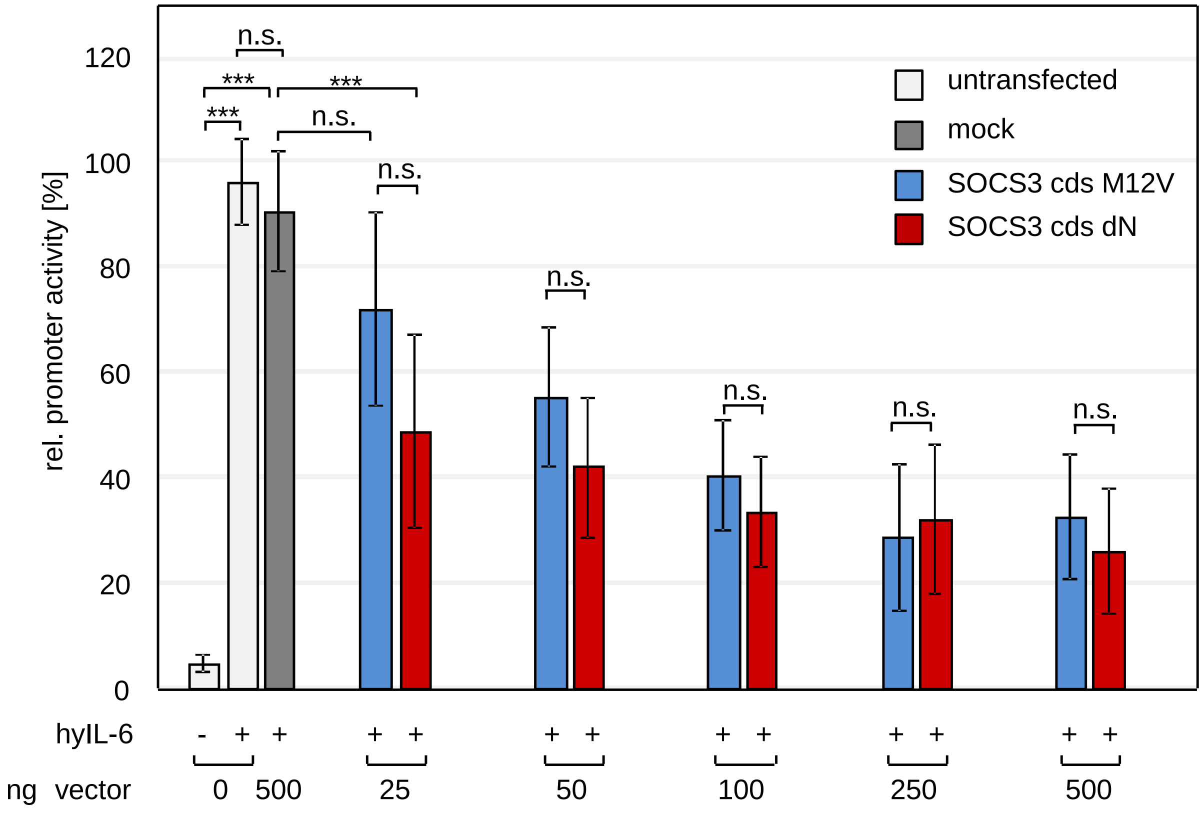

Supplementary Fig. S2. The inhibitory potential of SOCS3 does not depend on isoform-specific N-terminal peptide

HeLa cells were transfected with expression vectors for SOCS3 cds M12V or SOCS3 cds dN and expression vectors encoding a STAT3-reporter construct and a β-galactosidase encoding expression vector. Cells were treated for 16 h with hyIL-6 (10 ng/ml) to induce reporter gene expression. Luciferase activity was normalized to β-galactosidase activity. Maximal reporter activity was set to 100%. Data are given as mean of three independent experiments ± SD. rANOVA: *** $p < 0.001$, n.s. $p > 0.05$

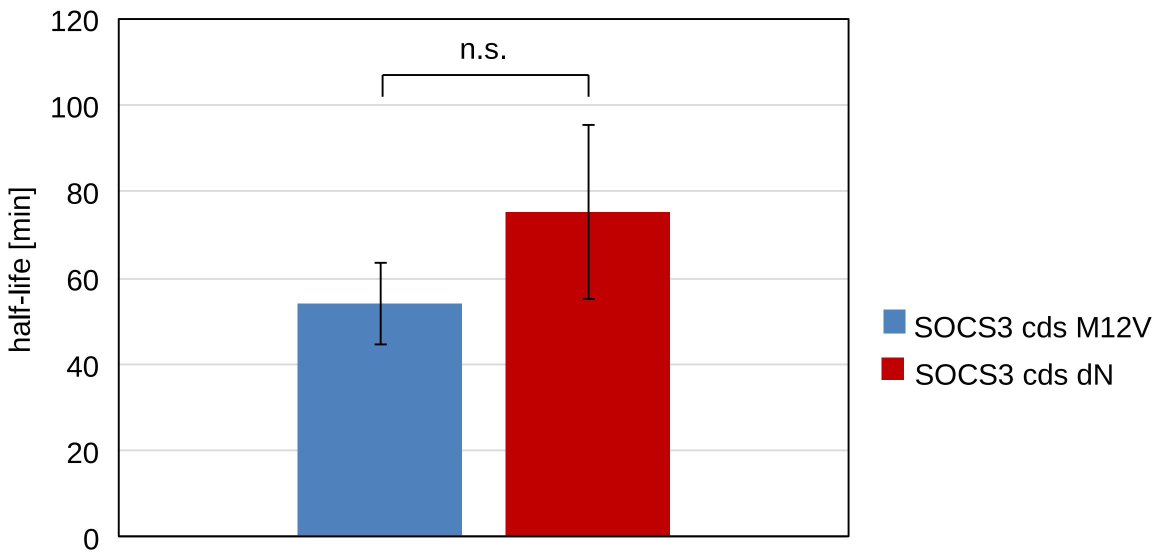

Supplementary Figure S3. Half-lives of SOCS3 isoforms in HEK293 cells

Quantification of the half-lives of the long (blue) and short (red) SOCS3 isoform from experiments as shown in Figure 3A. Expression was normalized to tubulin expression. Maximal SOCS3 protein expression was set to 100% for each specific data set. Calculation of the half-lives was performed by linear regression for each specific data set. Data are given as means of three independent experiments ± SD. rANOVA: n.s. $p > 0.05$

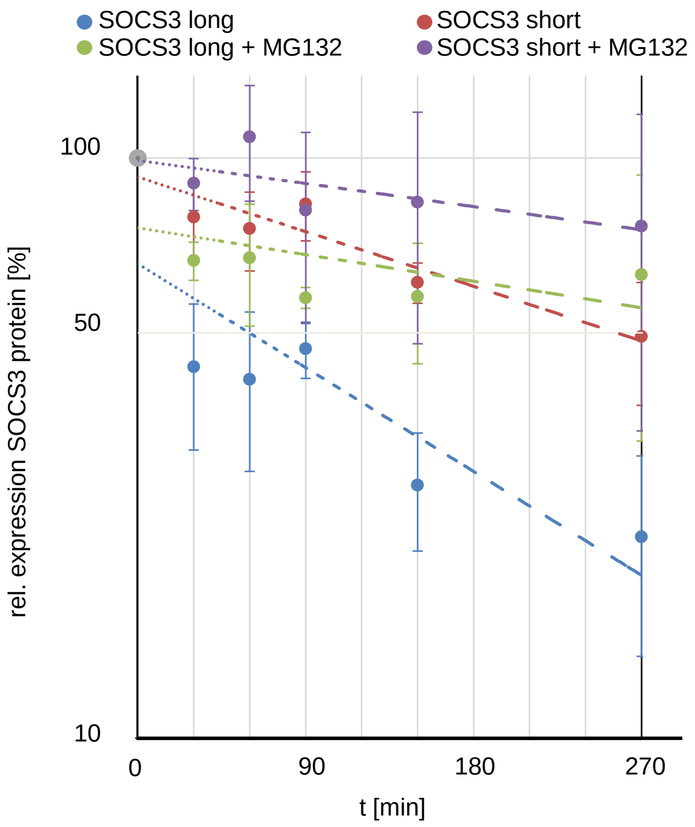

Supplementary Fig. S4. Quantification of the SOCS3 degradation

Quantification of the degradation of the long (blue) and short SOCS3 (red) isoform in the absence or presence of MG132 (green, purple, respectively) from experiments as shown in Figure 3B. Expression was normalized to tubulin expression. Maximal SOCS3 protein expression was set to 100% for each specific data set. Data are given as mean of three independent experiments ± SD. Linear regression was performed on averaged data. Half-lives are given in Figure 3C.

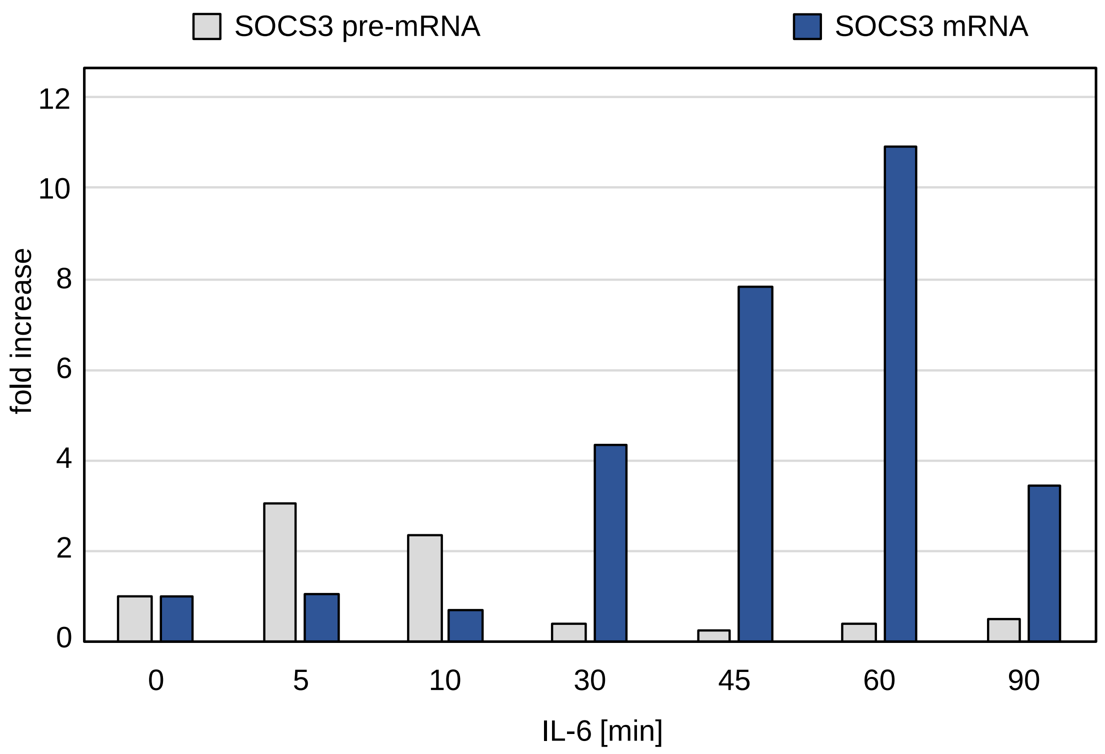

Supplementary Fig. S5. Quantification of *SOCS3* pre-mRNA and *SOCS3* mRNA

Quantification of SOCS3 pre-mRNA and SOCS3 mRNA expression as shown in Figure 4C. HepG2 cells were stimulated with 10 ng/ml IL-6 for the indicated times. mRNA was isolated, transcribed into cDNA and cDNA amplified as in Fig. 4B. Fold increase compared to the untreated cells is shown.

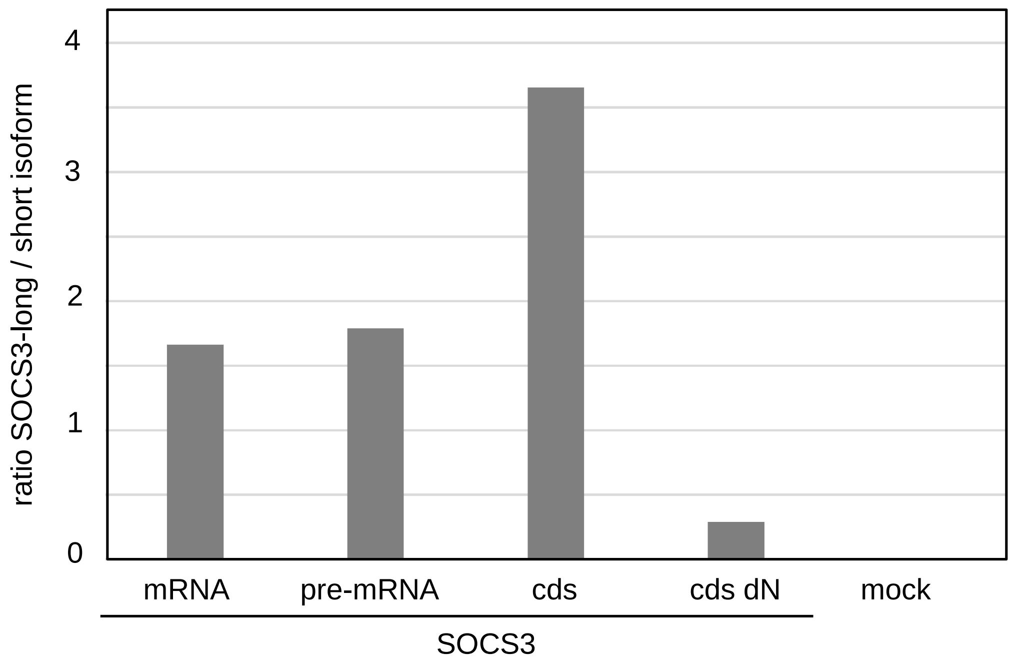

Supplementary Fig. S6. Quantification of SOCS3 isoform expression

Quantification of SOCS3 isoform expression shown in Figure 4D. HEK293 cells were transfected with expression vectors for SOCS3 pre-mRNA, SOCS3 mRNA, the coding sequence of SOCS3 (SOCS3 cds), and the coding sequence of the short isoform of SOCS3 (SOCS3 cds dN) (Fig. 4A). SOCS3 and tubulin protein expression was evaluated by Western blotting. Expression of the long isoform was normalized to expression of the short isoform.

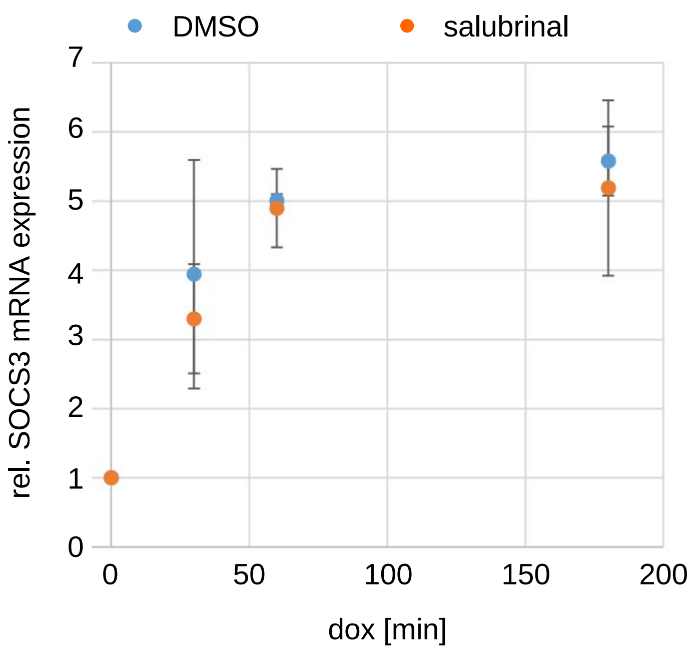

Supplementary Fig. S7. Salubrinal does not affect *SOCS3* mRNA expression

SOCS3 mRNA expression was induced by 1 µg/ml doxycycline in HEK293 FlpIn SOCS3 pre-mRNA cells for the times indicated. SOCS3 mRNA was quantified by qRT-PCR. Fold increase compared to the untreated cells is shown. Data are given as mean of three independent experiments ± SD.

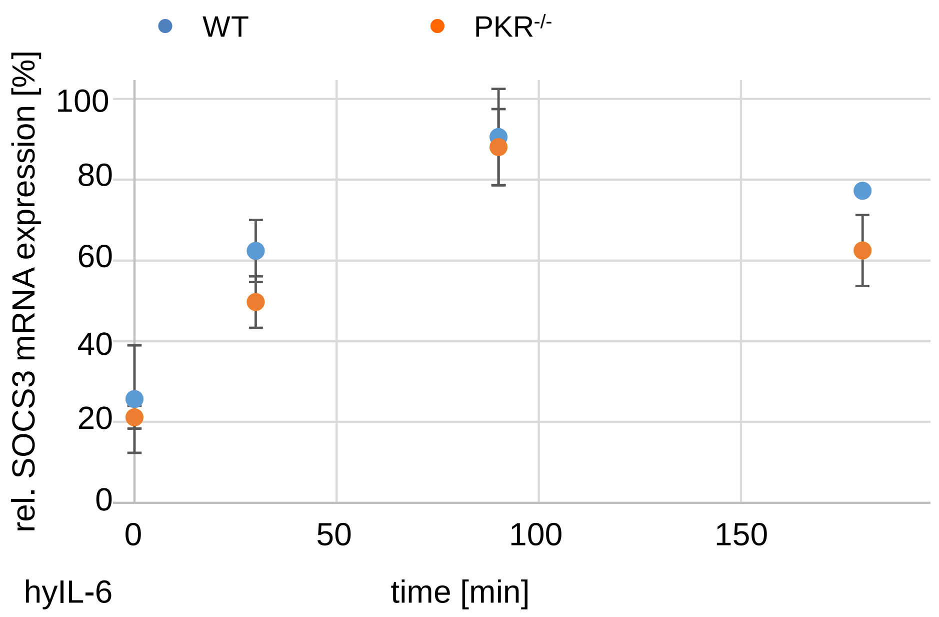

Supplementary Fig. S8. PKR knockout does not affect *SOCS3* mRNA expression

HEK293 cells and HEK293 PKR$^{-/-}$ cells were stimulated with 10 ng/ml hyIL-6 for the times indicated. Expression of SOCS3 mRNA was analysed using qRT-PCR. Maximal expression of SOCS3 mRNA was set to 100%. Data are given as mean of three independent experiments ± SD.

Human (*Homo sapiens*)	MVTHSKFPAAGMSRPLDTSLR------LKTFSSKSEYQLVVNAV
House mouse (*Mus musculus*)	MVTHSKFPAAGMSRPLDTSLR------LKTFSSKSEYQLVVNAV
Pig (*Sus scrofa*)	MVTHSKFPAAGMSRPLDTSLR------LKTFSSKSEYQLVVNAV
Koala (*Phascolarctos cinereus*)	MVTHSKFPATGMNRPLDTSLR------LKTFSSKSEYQLVVNAV
Chicken (*Gallus gallus*)	MVTHSKFPAAGMSRPLDTSLR------LKTFSSKSEYQLVVNTV
Three toed box turtle (*Terrapene mexicana*)	MVTHSKFPAAGMSRPLDTSLR------LKTFSSKSEYQLVVNAV
Tropical clawed frog (*Xenopus tropicalis*)	MVTQSKFPS--MSRALDSSLR------LKTFSSRSEYNLVLTAV
Zebrafish (*Danio rerio*)	MITHSKLDNA-MSSGLFDASMRLPFYHFKTFSSKVQFQLVQHTI
Rainbow trout (*Oncorhynchus mykiss*)	MVTHSKFDSV-MSSSPFDSTMRLPHRY-KTFSSKVQYQLVVTTL
Whale shark (*Rhincodon typus*)	MVTYSKFD--GMSRPVDVPAQRLSYR-LKTFSSKNEYNLIVNTV

Supplementary Fig. S9. SOCS3 protein-sequence homology at the N-terminal end

Alignment of the N-terminal protein sequences of SOCS3 found in diverse species. First and second translation initiation site are highlighted in green. Mismatches are indicated in red, while substitutions with amino acids with identical properties are indicated in blue.
